# Genome sequence of the English grain aphid, *Sitobion avenae* and its endosymbiont *Buchnera aphidicola*

**DOI:** 10.1093/g3journal/jkab418

**Published:** 2021-12-08

**Authors:** Stephen Byrne, Maximilian Schughart, James C Carolan, Michael Gaffney, Peter Thorpe, Gaynor Malloch, Tom Wilkinson, Louise McNamara

**Affiliations:** 1 Teagasc, Crop Science Department, Carlow R93 XE12, Ireland; 2 School of Biology and Environmental Science, University College Dublin, Dublin 4, Ireland; 3 Department of Biology, Maynooth University, Maynooth, Co. Kildare, W23 F2H6, Ireland; 4 Teagasc, Ashtown Research Centre, Dublin D15 KN3K, Ireland; 5 School of Medicine, University of St Andrews, North Haugh, KY16 9TF St Andrews, UK; 6 The James Hutton Institute, Invergowrie, Dundee DD2 5DA, UK

**Keywords:** aphid, PacBio HiFi, *Sitobion avenae*, genome

## Abstract

The English grain aphid, *Sitobion avenae*, is a major agricultural pest of wheat, barley and oats, and one of the principal vectors of barley yellow dwarf virus leading to significant reductions in grain yield, annually. Emerging resistance to and increasing regulation of insecticides has resulted in limited options for their control. Using PacBio HiFi data, we have produced a high-quality draft assembly of the *S. avenae* genome; generating a primary assembly with a total assembly size of 475.7 Mb, and an alternate assembly with a total assembly size of 430.8 Mb. Our primary assembly was highly contiguous with only 326 contigs and a contig N50 of 15.95 Mb. Assembly completeness was estimated at 97.7% using BUSCO analysis and 31,007 and 29,037 protein-coding genes were predicted from the primary and alternate assemblies, respectively. This assembly, which is to our knowledge the first for an insecticide resistant clonal lineage of English grain aphid, will provide novel insight into the molecular and mechanistic determinants of resistance and will facilitate future research into mechanisms of viral transmission and aphid behavior.

## Introduction

**Figure 1:**
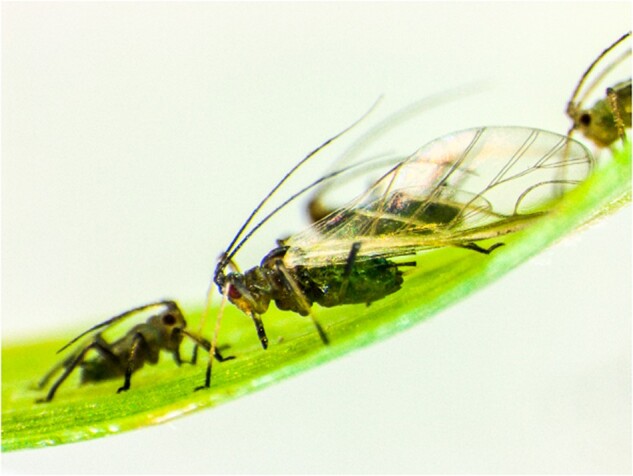
*Sitobion avenae* Fabricius (Hemiptera: Aphididae).

Aphids (Hemiptera: Aphidoidea) represent one of the most important insect pests of temperate agriculture. Aphid feeding reduces crop yield by removing photoassimilates, transmitting plant viruses, and altering plant growth and development. They typically reproduce by cyclical parthenogenesis, a reproductive mode which alternates between a single sexual generation produced in autumn (with decreasing photoperiod and temperature) and several clonal generations produced during spring/summer conditions (Dedryver *et al.* 2005). The English grain aphid, *Sitobion avenae* Fabricius (Hemiptera: Aphididae), is one of the most destructive cereal aphids in Western Europe, feeding on cereals including barley, wheat, and rice (Figure 1).

The English grain aphid is a major vector of barley yellow dwarf virus (BYDV), an economically important plant virus. The primary method of *S.avenae* control is through the application of pyrethroid insecticides. However, over the past decade pyrethroid resistant clones have been frequently detected across Great Britain and Ireland. The most common resistance mechanism is termed knockdown resistance (kdr/super-kdr) and results from mutation of the voltage gated sodium channel (VGSC) gene, leading to amino acid substitutions within the channel protein that reduce the sensitivity to the specific pyrethroid (Foster *et al.* 2014).

Resistant *S. avenae* grain aphids were initially thought to be derived from a single, dominant, and established SA3 clone in England and Scotland (Malloch *et al.* 2016), which is sometimes referred to as a super-clone (Walsh *et al.* 2019), particularly when it becomes the prevalent clone within the overall species population. In a survey of kdr aphids conducted in Ireland in 2016, the dominant resistant clone detected in the kdr-SR (heterozygote) genotype was also the SA3 clone (Walsh *et al.* 2020). The kdr-heterozygote SA3 clone can survive pyrethroid contact, and can continually reproduce parthenogenetically under laboratory conditions at a comparable rate to the unexposed individuals of the susceptible (kdr-SS) SA27 clone (Walsh *et al.* 2019). Therefore, the SA3 clone of *S. avenae* presents an interesting case for further studies involving insecticide resistance and viral transmission in agronomically important aphids.

In recent years, a number of annotated genomes have been produced for species within the subfamily Aphidinae; including *Acyrthosiphon pisum* (pea aphid) (IAGC 2010), *Pentalonia nigronervosa* (banana aphid) (Mathers *et al.* 2020), *Myzus persicae* (green peach aphid) (Mathers *et al.* 2021), *Diuraphis noxia* (Russian wheat aphid) (Burger and Botha 2017), *Rhopalosiphum maidis* (corn leaf aphid) (Chen *et al.* 2019), *Myzus cerasi* (black cherry aphid), and *Rhopalosiphum padi* (bird cherry oat aphid) (Thorpe *et al.* 2018), and *Sitobion miscanthi* (Indian grain aphid) (Jiang *et al.* 2019). However, no genome has been published to date for *S.avenae*, despite it being a major agricultural pest of wheat, barley and oats, and a major vector of BYDV.

To date, assemblies of aphid genomes have focused on collapsing divergent alleles and producing a consensus sequence of both haplotypes. While highly accurate short reads are suitable for characterizing genetic variation, and estimating transcript abundance, longer reads are generally preferable for de novo genome assembly applications. Although longer reads can suffer from lower accuracies which may impact assembly continuity and accuracy, the latest approaches to produce long reads (15–25 Kb) with high accuracy (Wenger *et al.* 2019) now offer opportunities to separate highly similar repeats and alleles, as was recently demonstrated (Nurk *et al.* 2020). A recent technical evaluation of assembly strategies in barley concluded that assemblies generated with HiFi data reach a level of contiguity previously only achievable with a complex process of iterative scaffolding (Mascher *et al.* 2021).

Here, we report the de novo genome sequence of the grain aphid, *S.avenae* SA3 clone and its endosymbiont *Buchnera aphidicola* using Pacific Biosciences (PacBio) HiFi sequencing data. On the basis of a 475.7 Mb primary assembly consisting of 326 contigs, we identified 31,007 protein-coding genes. We have also produced an alternate assembly of 434.1 Mb, representing alternate alleles and predicted 29,037 protein-coding genes. The *S. avenae* SA3 genome will lead to a better understanding of insecticide resistance and facilitate further research into aphid behavior and virus transmission in this agriculturally important species.

## Materials and methods

### Insect rearing, DNA isolation, and sequencing

A laboratory maintained colony of asexually reproducing *S. avenae*, originating from a single grain aphid was used for all DNA and RNA extractions in this study. The maternal grain aphid was collected in 2017 from a wheat field in Carlow, Ireland, and removed from the colony and stored after producing nymphs. The colony was maintained on barley plants in an insect incubator at 20°C and 16/8 h day-night cycle. Genotyping of the original maternal aphid and aphids from following generations confirmed that the aphids were of the SA3 clonal lineage, which has partial resistance to pyrethroid (Foster *et al.* 2014).

High molecular weight (HMW) DNA was extracted using the Nanobind Big DNA Kit (Circulomics, Baltimore, USA), following a manufacturers protocol [High molecular weight insect DNA extraction Protocol v0.18, 1/2020, Circulomics; Protocol 1: HMW (50 kb 400+ kb) DNA extraction from *Drosophila* species homogenized with pellet pestle] with some minor modifications. For the extractions, a bulk collection of 25 mg live *S. avenae* SA3 aphids was homogenized in a 1.5 ml microcentrifuge tube using 500 l cold Buffer CT and a sterilized plastic pestle. The quantity of DNA was estimated with a NanoDrop Spectrophotometer (Thermo Fisher Scientific, Waltham, MA, USA) and successful extraction of HMW-DNA was confirmed using agarose gel electrophoresis. Short read DNA sequencing (PE 150 bp) was carried out with an Illumina NovaSeq (NovoGene, UK) and over 26.2 Gbp of sequence data was generated. HMW-DNA was then sequenced using PacBio CCS technology to generate 31.98 Gbp of HiFi reads with an average length of 11.7 Kb.

### Transcriptome sequencing and quantification

To support the *ab initio* gene predictions RNA sequencing was conducted for a total of 24 *S. avenae* (SA3-clone) samples (6X winged-heads, 6X winged-bodies, 6X unwinged-aphids, and 6X winged-aphids). Each sample consisted of 50 individual aphids, which were transferred to a 1.5 ml RNAse free micro-centrifuge tube containing 500 l Lysis Buffer (10 l 2-Mercaptoethanol + 500 l Lysis Solution). The aphids were homogenized using a plastic pestle and a motorized hand drill. The homogenate was snap-frozen in liquid nitrogen and homogenized again with the pestle. This step was repeated three times in total. RNA extraction was conducted using the GenElute Mammalian Total RNA Miniprep Kit (Sigma-Aldrich; Merck KGaA, Darmstadt, Germany), with minor modifications with each extraction comprising 50 aphids per sample. For the head and body samples, 50 live aphids were transferred to a glass microscope slide holding a droplet of lysis buffer, where the heads and bodies were separated by dissecting in front of the thorax using a sterile scalpel. Heads and bodies were pooled separately into 1.5 ml RNAse free micro-centrifuge tubes containing 500 l Lysis Buffer. Homogenization and RNA extraction was conducted following the same steps as the whole bodies. RNA was quantified with a NanoDrop Spectrophotometer (Thermo Fisher Scientific, Waltham, MA, USA) and the quality was assessed using agarose gel electrophoresis.

Strand-specific mRNA libraries were prepared and sequenced with DNBseq (BGI, Hong Kong) to generate a minimum of 30 million paired reads (150 bp) per sample. Raw reads were filtered to remove adaptor sequences, contamination, and low-quality reads; leaving between 32.9 and 35.5 million read pairs per sample.

### Genome assembly

The genome size of *S. avenae* was estimated using k-mer analysis; a k-mer (*k* = 21) distribution was generated with Jellyfish (v2.2.10) (Marçais and Kingsford 2011) using the HiFi reads and genome characteristics generated with GenomeScope (Vurture *et al.* 2017).

The *S. avenae* PacBio HiFi data consisted of 31.98 Gb with a mean read length of 11.7 Kb, which was assembled using hifiasm (0.14-r312) (Cheng *et al.* 2021) with default parameters. The completeness of the resulting primary and alternate assemblies were assessed using Benchmarking Universal Single-Copy Orthologs (BUSCO) version 3.0.2 (Simão *et al.* 2015) run in genome mode and with the lineage dataset Arthropoda odb9 (consisting of 1066 BUSCO groups). We also used the K-mer Analysis Toolkit (KAT) (v2.4.2) (Mapleson *et al.* 2017) to generate assembly spectra copy number plots (spectra-cn) to check assembly coherence against reads used to generate assembly.

The primary and alternative assemblies were both screened for contaminants using the BlobTools pipeline (v1.1.1) (Laetsch and Blaxter 2017). Illumina data were aligned to each assembly using bwa (Li 2013), contigs were given a taxonomic assignment based on blastn results against NCBI nt database, and plots were generated showing contig GC content, coverage, and taxonomic assignment. The plots and results were used to remove non-Hemiptera contigs and likely contaminants from both primary and alternate assemblies.

### Repeat masking

A de novo repeat library was generated using RepeatModeler (v2.0.1) (Flynn *et al.* 2020), integrating results from RepeatScout (Price *et al.* 2005), TRF (Benson 1999), and RECON Bao and Eddy (2002). The resulting repeats were filtered to remove repeats that are part of protein-coding genes. A protein set was downloaded from AphidBase (aphidbase-2.1b-pep.fasta) and TransposonPSI (http://transposonpsi.sourceforge.net) was used to identify potential transposon ORFs, and the protein set was filtered to remove proteins with sequence homology to transposable elements. The repeats from RepeatModeler were used in a blastx search against the filtered protein set, and hits were removed from our RepeatModeler library to generate a final repeat library for use in repeat masking. RepeatMasker was configured with the complete Dfam library (version 3.2), and was used with the de novo filtered repeat library to identify and soft-mask repeats in the assemblies prior to annotation.

### Genome annotation

Structural annotation of the genome was carried out using the BRAKER2 pipeline (v2.1.5) (Stanke *et al.* 2006, 2008; Li *et al.* 2009; Barnett *et al.* 2011; Lomsadze *et al.* 2014; Buchfink *et al.* 2015; Hoff *et al.* 2016, 2019). The pipeline was used with 24 strand-specific RNAseq libraries that were aligned to the soft-masked genome using HISAT2 (Kim *et al.* 2019) to provide hints, which were then used with GenMark-ET to predict genes and retrain Augustus.

Functional annotation of predicted proteins was carried out using InterProScan (v5.51-85.0) (Jones *et al.* 2014; Blum *et al.* 2021) to search for protein domains, motifs, and signatures using publicly available databases.

RNA-seq data were pseudoaligned to the predicted transcriptome and transcript abundance determined using Kallisto (Bray *et al.* 2016). Differential gene expression was carried out using Sleuth (Pimentel *et al.* 2017) with two pairwise comparisons; heads compared to bodies, and winged compared to unwinged. To identify the transcripts differentially expressed we ran a likelihood ratio test (LRT), followed by a Wald test to determine *β* estimates and only transcripts overlapping with the LRT analysis were retained.

### Orthology and phylogenomic analysis

The protein sequences from our *S.avenae* assembly and nine previously published aphid genomes (*Aphis glycines*, *A.pisum*, *Cinara cedri*, *D.noxia*, *M.cerasi*, *M.persicae*, *P.nigronervosa*, *R.maidis*, and *R.padi*) were clustered into orthogroups using OrthoFinder v2.5.4 (Emms and Kelly 2015, 2019). Input sequence data for OrthoFinder consisted of the longest transcript at each gene, downloaded from https://doi.org/10.5281/zenodo.3765644 for previously published aphid genomes (Mathers *et al.* 2020). OrthoFinder was run using the multiple sequence alignment (MSA) method for tree inference (Price *et al.* 2010; Emms and Kelly 2017, 2018) (-M msa -S diamond -T fastree), conferring species tree from a concatenated MSA of single copy genes. The species tree was visualized using Dendroscope (Huson *et al.* 2007).

A recent study quantified gene numbers for detoxification-related genes in seven aphid species from the Macrosiphini and Aphidini tribes (Chen *et al.* 2019). We have updated this with inclusion of gene numbers from *S.avenae* (this study), and the recently sequenced banana aphid, *P.nigronervosa* (Mathers *et al.* 2020). In line with the previous study (Chen *et al.* 2019), we identified genes based on protein domains predicted through InterProScan; cytochrome P450 genes(InterPro ID: IPR001128), glutathione S-transferases (GSTs) (InterPro ID: IPR004045, IPR004046), carboxylesterases (InterPro domain ID: IPR002018), UDP-glucuronosyltransferases (InterPro domain ID: IPR002213), and ABC transporters (InterPro ID: IPR003439).

### Endosymbiont assembly and annotation

The hifiasm assembly generated 937 contigs with the taxonomic assignment Enterobacterales, a total sum of 21.25 Mb, and an N50 of 22.6 Kb. It was suspected that much of these contigs originate from *B.aphidicola*. In order to generate a more contiguous assembly of the *B.aphidicola* genome, we first mapped HiFi data to the primary assembly using bwa (Li 2013), created a bed file of non-Hemiptera contigs, and used samtools (Li *et al.* 2009) to extract those reads that mapped to non-Hemiptera contigs. The de novo assembler Flye (v2.8) (Kolmogorov *et al.* 2020) was then used to assemble these reads (with options; –pacbio-hifi, and –meta). The *B.aphidicola* genome was identified from this assembly and annotated using prokka (Seemann 2014). The annotated *B.aphidicola* genome was visualized using the python package genome diagram (Pritchard *et al.* 2006).

## Results and discussion

### The *S. avenae* genome characteristics

**Figure 2:**
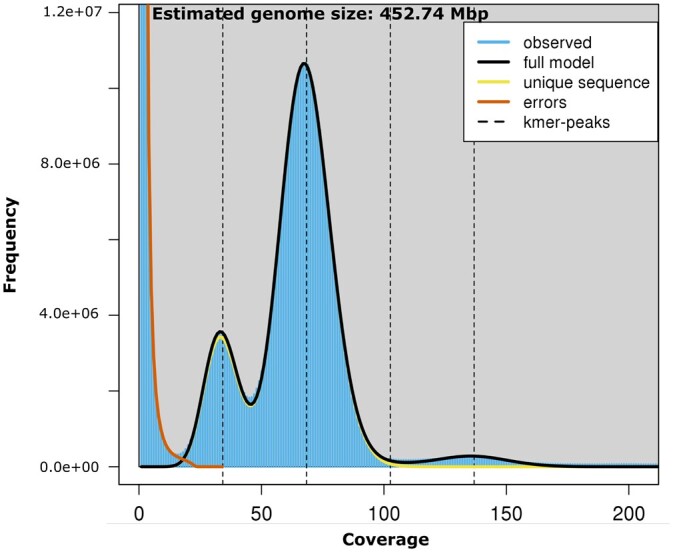
A K-mer (*K* = 21) distribution based on the high coverage HiFi reads as modeled and visualized by genomescope. The first and second dotted lines correspond to the peak of the heterozygous and homozygous portion of the genome, respectively. The third and fourth dotted lines correspond to the duplicated heterozygous and homozygous regions, respectively.

The size of the *S. avenae* genome was estimated to be 452.7 Mb (Figure 2) using k-mer analysis (*k* = 21) based on the HiFi sequence data. Using this estimate we have generated over a 70-fold sequence coverage of the genome with PacBio HiFi data. This is comparable to a genome size estimate of 431.1 Mb for *S. avenae* that was determined by flow cytometry (Wenger *et al.* 2020), and where the average genome size of 19 aphid species was 464.4 ± 21.4 Mb. As a comparison, the genome assembly size of the closely related Indian grain aphid *S.miscanthi* was 397.9 Mb (Jiang *et al.* 2019). The HiFi data set released as part of this study represents the first publicly available deep coverage HiFi data set for an aphid species, and complements recently released HiFi data of four complex plant and animal genomes and a mock metagenome (Hon *et al.* 2020),

The genome repeat length was estimated to be 33.7% and is in line with repeat content for a range of other aphid species including *S. miscanthi*, where total repeat content was estimated at 31.15% (Jiang *et al.* 2019).

### *Sitobion avenae* genome assembly and completeness

**Figure 3:**
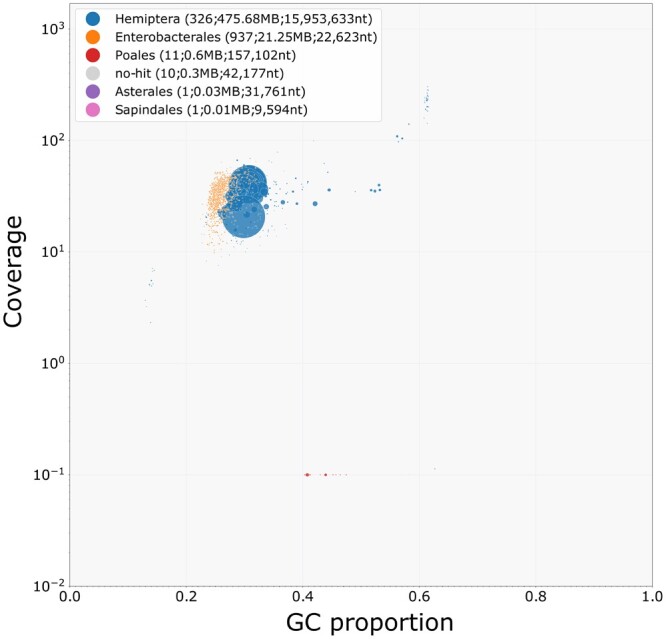
A taxon-annotated GC proportion and coverage plot of the *S. avenae* genome. Each bubble represents an assembled contig and bubble size is linked to contg size, and bubbles are colored according to taxon annotation. The many small orange bubbles correspond to *B. aphidicola* contigs with a total length of 21.25 Mb, and the predominantly larger blue bubbles correspond to Hemiptera contigs with a total length of 475.68 Mb.

A de novo assembly of the PacBio HiFi data with hifiasm produced a primary assembly of 497.9 Mb and an alternate assembly of 434.1 Mb. We evaluated the assembly for contamination using plots of GC proportion of contigs (with taxonomic assignment) against coverage with Illumina short reads (Figure 3). Encouragingly, 98.76% of the Illumina reads mapped to the HiFi primary assembly, and 94.57% of the Illumina reads mapped to the HiFi alternate assembly.

**Figure 4:**
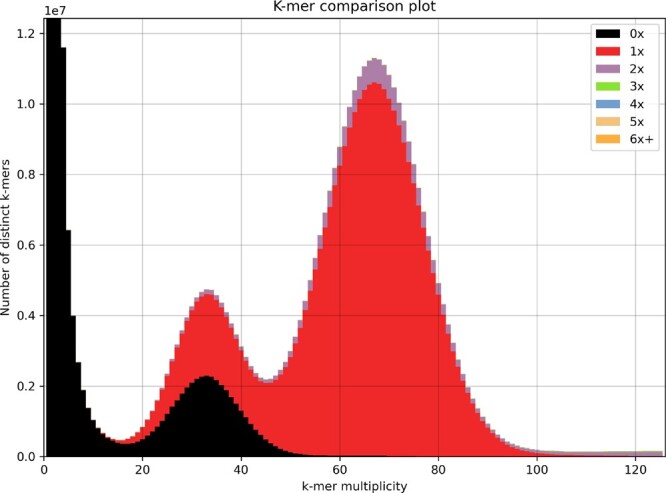
KAT k-mer spectra plot comparing k-mer content of HiFi reads to k-mer content of final primary assembly (after removing non-Hemiptera contigs). The first peak represents the heterozygous content and the second peak the homozygous content. The black peak represents lost content and represents approximately half the heterozygous content (47.89%). The amount of duplicated content is 5.29% of the homozygous peak and 3.60% of the heterozygous peak.

As expected, the majority of large contigs were assigned as Hemiptera, making up 475.68 Mb of the total assembly length. However, the majority of contigs (937) in the assembly were assigned as Enterobacterales, making up 21.25 Mb of the total assembly length and likely originating from *B.aphidicola*. All non-Hemiptera contigs were removed, giving a final primary assembly of 475.7 Mb and an alternate assembly of 430.8 Mb. An analysis of the final primary genome assembly using k-mer spectra predominantly shows a single haplotype mosaic with each heterozygous region represented once in the assembly (Figure 4). The lost content in the heterozygous peak represents half the heterozygous content, which will have been captured in the alternate assembly.

The final primary assembly consisted of 326 contigs with an N50 of 15.95 Mb (Table 1), and represents good continuity when compared to other published aphid genomes; for example, *P.nigronervosa* with 20,873 contigs and a contig N50 of 64.04 Kb (Mathers *et al.* 2020), *M.cerasi* with 49,349 scaffolds and a scaffold N50 of 23.3 Kb (Thorpe *et al.* 2018), *R.padi* with 15,615 scaffolds and a scaffold N50 of 116.2 Kb (Thorpe *et al.* 2018). However, the above assemblies were all generated using short-read data and a more relevant comparison would be to two recent aphid assemblies, *R.maidis* and *S.miscanthi*, generated using PacBio long-read sequencing (continuous long reads as opposed to the CCS/HiFi data generated in the current study). In both cases collapsed assemblies representing a single haplotype mosaic were generated; in the case of *R.maidis* the assembly consisted of 689 contigs with a contig N50 of 9.1 Mb, and in the case of *S.miscanthi* the assembly consisted of 1148 contigs with a contig N50 of 1.6 Mb. Further scaffolding of both assemblies using chromosome conformation capture methods generated chromosome level scaffolds, and while further scaffolding is outside the scope of the current study, it is anticipated that similar approaches would generate chromosome-scale assemblies given our highly contiguous contig assembly as a starting point.

**Table 1:** Statistics of the *S. avenae* genome assembly

	Primary contigs^*a*^	Alternate contigs^*b*^
Total assembly size (Mb)	475.7	430.8
Number of contigs	326	3,139
N50 contig length (Mb)	15.95	0.36
Max contig length (Mb)	36.43	2.14
Min contig length (bp)	4,802	10,063
Complete BUSCOs	97.7% (1,042)	93.8% (1,000)
Complete and single copy BUSCOs	93.8% (1,000)	87.4% (932)
Complete and duplicated BUSCOs	3.9% (42)	6.4% (68)
Fragmented BUSCOs	0.4% (4)	1.1% (12)
Missing BUSCOs	1.9% (20)	5.1% (54)
Protein coding genes (*n*)	31,007	29,037
Transcripts (*n*)	33,389	31,259
Mean CDS length (bp)	1,231	1,188
Mean exons per mRNA (*n*)	5	5
Total CDS length (Mb)	41.1	37.1

a Contigs including one set of haplotypes after filtering non-Hemiptera contigs.

b Assembly consisting of alternate alleles of contigs present in the primary assembly after filtering non-Hemiptera contigs.

To assess genome completeness a BUSCO analysis using an Arthropoda lineage data set was conducted. This analysis involves the identification of conserved single-copy, orthologous genes, and enabled a determination of completeness and level of duplication in both primary and alternate assemblies. In the primary assembly, 97.7% of BUSCOs were present and complete and 3.9% were duplicated, compared to 93.8% of BUSCOs complete and 6.4% duplicated in the alternate assembly (Table 1). The high levels of completeness in both assemblies and low levels of duplicated BUSCOs support the assessment that alleles from both haplotypes have been comprehensively captured, and that these have been successfully partitioned into two assemblies.

### *Sitobion avenae* annotation and transcript quantification

We identified and soft-masked 31.88% of the primary genome assembly, which was in line with our estimates of repeat content from k-mer analysis and the repeat content of *S.miscanthi* (31.15) (Jiang *et al.* 2019). Structural annotation of the masked genome was carried out using the BRAKER2 pipeline with over 247 Gbp of RNAseq evidence, generated from 24 strand-specific libraries. This resulted in the annotation of 31,007 protein-coding genes and 33,389 transcripts, a mean exon length of 224 bp, and with the total length of exons accounting for just over 41 Mbp (8.6%) of the genome (Table 1). The number of predicted genes is similar to what has been reported for many aphid species (IAGC 2010; Burger and Botha 2017; Thorpe *et al.* 2018; Mathers *et al.* 2020); although is approximately double the number of genes recently predicted in an assembly of the *S.miscanthi* genome (Jiang *et al.* 2019). We also carried out annotation of the alternate assembly using the same strategy and predicted 29,037 genes with similar metrics to the primary annotation (Table 1). Functional annotation of predicted proteins was carried out using InterProScan (v5.51-85.0) (Jones *et al.* 2014; Blum *et al.* 2021) to search for protein domains, motifs, and signatures using publicly available databases (see Supplementary material with functional annotation).

**Figure 5:**
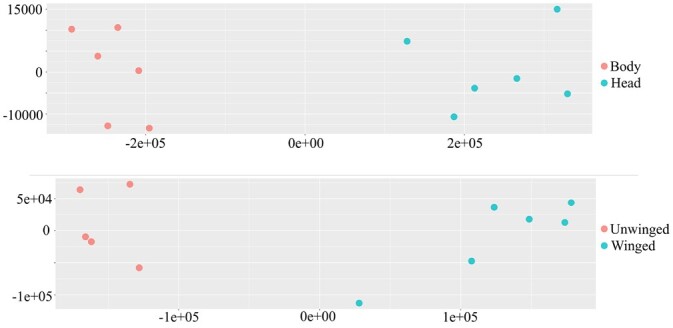
PCA of transcript abundance based on body and head samples (top plot) and winged and unwinged aphids (bottom plot). The horizontal axis represents the first principal component and the vertical axis represents the second principal component.

Sequence data from the 24 strand-specific RNAseq libraries were pseudolaligned to the above annotations (33,389 transcripts) and transcript abundance estimated. An initial PCA identified a potential sample mix-up, where an unwinged sample was labeled as winged and this sample was therefore removed from further analysis. PCA with estimated counts clearly separated samples on the first principal component according to body/head and winged/unwinged (Figure 5), with PC1 explaining over 75% of the variance in both cases. The fraction of reads aligned to the reference transcriptome in all cases was approximately 42%; alignment rates to the genome with HiSat2 were over 85%. Examples of alignment rates to predicted transcripts in other aphid species ranged between 49% and 64% (Thorpe *et al.* 2018). We identified 8660 transcripts differentially expressed between head and body samples, and 3133 transcripts differentially expressed between winged and unwinged samples, and sleuth objects associated with this analysis are available as Supplementary data (winged/unwinged, and https://doi.org/10.6084/m9.figshare.14797557).

### Phylogenomic analysis

**Figure 6:**
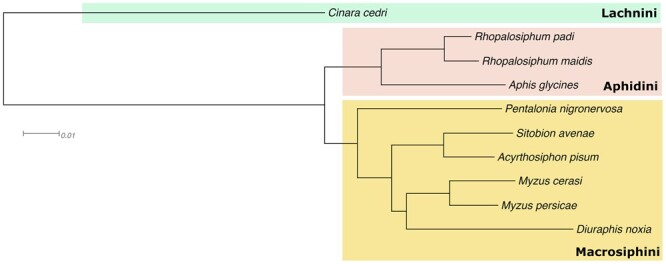
Maximum likelihood phylogeny of aphid species included in Mathers *et al.* (2020) with addition of *S. avenae* from this study. The species tree was inferred from a concatenated multiple sequence alignment of 4249 single copy genes present in all species and there was full support at all branches. Branch lengths are in amino acid substitutions per site.

We carried out orthology clustering of our predicted proteins from *S. avenae* and nine other aphid species. OrthoFinder assigned 234,168 genes (91.9%) to 23,797 orthogroups (see Supplementary File). A total of 7497 orthogroups were shared by all 10 species present, including 4249 single-copy genes present in all species (see Supplementary material). The percentage of *S. avenae* genes in orthogroups was 91.9%, including 510 *S. avenae* -specific orthogroups. Complete orthogroup data for the comparative analysis is available as Supplementary data. A species tree was inferred from a concatenated multiple sequence alignment of the 4249 one-to-one orthologs (Figure 6). The species tree identified that *S. avenae* was close to the pea aphid (*A. pisum*), which confirms phylogeny established from fragments of mitochondrial cytochrome oxidase I and 12S rRNA genes (Papasotiropoulos *et al.* 2013).

### Detoxification and insecticide resistance

**Figure 7:**
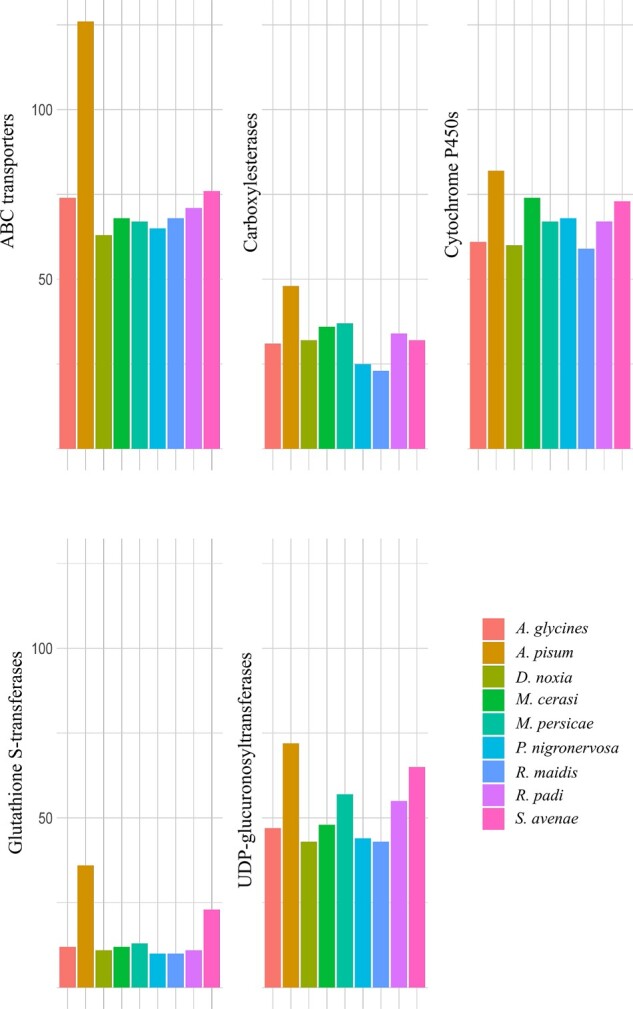
Gene numbers in Aphidini and Macrosiphini species identified as ABC transporters (InterPro ID: IPR003439), carboxylesterases (InterPro domain ID: IPR002018), cytochrome P450s (InterPro ID: IPR001128), glutathione S-transferases (InterPro ID: IPR004045, IPR004046), and UDP-glucuronosyltransferases (InterPro domain ID: IPR002213). These genes have been linked with detoxification and insecticide resistance.

Several gene families have been implicated in metabolic resistance to plant defense compounds and insecticides (reviewed in Li *et al.* 2007). These include the cytochrome P450 monooxygenases (P450s), carboxylesterases, UDP-glucuronosyltransferases, glutathione-S-transferases (GSTs), and ABC transporters. The number of detoxification-related genes was recently quantified for seven aphid species (Chen *et al.* 2019), and we have updated this with numbers from *P. nigronervosa* (Mathers *et al.* 2020) and *S. avenae* (Figure 7).

The greatest number of detoxification genes was present in *A. pisum*, followed by *S. avenae*, which is the species most closely related to *A. pisum* on the phylogenetic species tree shown in Figure 6. In particular, relatively high numbers of GSTs (23) and UDP-glucuronosyltransferases (65) were identified in *S. avenae* (Figure 7). GSTs have been implicated in detoxification of pyrethroids; for example, GSTs conferred resistance to pyrethroids in the brown planthopper *Nilaparvata lugens* by attenuating pyrethroid-induced lipid peroxidation (Vontas *et al.* 2001), and similarly a UDP-glucuronosyltransferases was shown to play a role in detoxification of pesticides by eliminating oxidative stress in *Apis cerana* (Cui *et al.* 2020). Of the 23 GST genes identified in *S. avenae*, two were placed in an *S. avenae* specific orthogroup, one was not placed in any orthogroup, eight were placed in five *A. pisum* and *S. avenae* specific orthogroups, and the remaining eleven in orthogroups with genes from multiple aphid species. Studies indicate that gene duplication may be widely used mechanisms of adaptive evolution in aphid species (reviewed in Bass and Field 2011).

### *Buchnera aphidicola* assembly and annotation

We extracted reads that did not map to Hemiptera contigs and assembled these reads using flye, which resulted in the assembly of a circular contig of 637,558 bp (see Supplementary Figure) with over 2500 times coverage and sharing the greatest sequence homology with the obligate endosymbiont *B.aphidicola*.

Annotation of this genome with prokka identified three rRNA, 32 tRNA, and 572 CDS. The most recent shared *Buchnera* ancestor possessed 616 protein-coding genes, and a recent study found that 257 of these genes were present in all strains of a diverse collection of *Buchnera*. Interestingly, 359 genes were missing in at least one of the 39 genomes; leading to genomes ranging in length from 412 to 646 Kb and containing anywhere from 354 to 587 protein-coding genes (Chong *et al.* 2019). A *Buchnera* strain from *S. avenae* was included in the above study and CDS number (572) was identical, and genome length (636,177) comparable to the *Buchnera* genome sequenced in the current study.

## Conclusion

Using PacBio HiFi data, we have produced, to our knowledge, the first draft assembly of the *S.avenae* genome; generating a primary assembly with a total assembly size of 475.7 Mb, and an alternate assembly with a total assembly size of 430.8 Mb. Our primary assembly had high continuity with only 326 contigs and a contig N50 of 15.95 Mb, and a completeness of 97.7% as estimated using BUSCO analysis. We predicted 31,007 protein-coding genes in the primary assembly and 29,037 in the alternate assembly. This assembly of a strain with partial resistance to insecticides will facilitate research into mechanisms of resistance, and help promote future research into mechanisms of aphid behavior and virus transmission.

## Data availability

A BioProject with accession number PRJNA730105 has been created on NCBI and both Illumina and PacBio HiFi data have been deposited (SRR14554260 and SRR14566019). The primary, alternate, and endosymbiont assemblies, associated GFF files and functional annotation, orthology analysis, and supplementary diagrams have been deposited in the following collection on Figshare (see grain aphid genomics, https://doi.org/10.6084/m9.figshare.c.5425896.v1). The RNA sequencing data have been deposited in ArrayExpress with accession number E-MTAB-10540, and data objects associated with differential expression analysis have deposited on Figshare (see grain aphid genomics).
